# Endoscopic Resection of Ampullary Tumours: Long-term Outcomes and Adverse Events

**DOI:** 10.1093/jcag/gwz007

**Published:** 2019-03-18

**Authors:** Ali Alali, Alberto Espino, Maria Moris, Myriam Martel, Ingrid Schwartz, Maria Cirocco, Catherine Streutker, Jeffrey Mosko, Paul Kortan, Alan Barkun, Gary R May

**Affiliations:** 1 The Center for Therapeutic Endoscopy and Endoscopic Oncology, St. Michael’s Hospital, University of Toronto, Toronto, Ontario, Canada; 2 Haya Al-Habeeb Gastroenterology and Hepatology Center, Mubarak Al-Kabeer Hospital, Jabriya, Kuwait; 3 Department of Gastroenterology, Pontificia Universidad Católica de Chile, Endoscopy Unit Hospital UC-Christus, Santiago, Chile; 4 Division of Gastroenterology, McGill University Health Center, McGill University, Montreal, Quebec, Canada

**Keywords:** *Adenoma*, *Ampulla*, *Ampullectomy*, *ERCP*, *Polypectomy*

## Abstract

**Background:**

The management of ampullary lesions has shifted from surgical approach to endoscopic resection. Previous reports were limited by small numbers of patients and short follow-up. The aim of this study is to describe short- and long-term outcomes in a large cohort of patients undergoing endoscopic ampullectomy.

**Methods:**

Retrospective study of endoscopic ampullectomies performed at a tertiary center from January 1999 to October 2016. Information recorded includes patient demographics, clinical outcomes, lesion pathology, procedural events, adverse events and follow-up data.

**Results:**

Overall, 103 patients underwent endoscopic resection of ampullary tumours (mean age 62.3 ± 14.3 years, 50.5% female, mean lesion size 20.9 mm; 94.9% adenomas, with a majority of lesions exhibiting low-grade dysplasia (72.7%). Complete endoscopic resection was achieved in 82.5% at initial procedure. Final complete endoscopic resection was achieved in all patients with benign pathology on follow-up procedures. Final pathology showed that 11% had previously undiagnosed invasive carcinoma. Delayed postprocedure bleeding occurred in 21.4%, all of which were managed successfully at endoscopy. Acute pancreatitis complicated 15.5% of procedures (mild in 93.8%). Perforation occurred in 5.8%, all treated conservatively except for one patient requiring surgery. Piecemeal resection was associated with significantly higher recurrence compared to en-bloc resection (54.3% versus 26.2%, respectively, *P* = 0.012). All recurrences were treated endoscopically.

**Conclusion:**

Endoscopic ampullectomy appears both safe and effective in managing patients with ampullary tumours in experienced hands. Most adverse events can be managed conservatively. Many patients develop recurrence during long-term follow-up but can be managed endoscopically. Recurrence rates may be reduced by performing initial en-bloc resection.

Tumours of the major duodenal papilla are rare, with an approximate 5% incidence of all gastrointestinal neoplasms, but are being identified more frequently with increasing numbers of endoscopies being performed (1,2). There are different types, including adenomas, adenocarcinomas, neuroendocrine tumours, lipomas and hamartomas (3,4). Adenomas are most frequently encountered; autopsy series have estimated the prevalence of ampullary adenoma to be 0.04% to 0.12% (5,6). Ampullary adenomas may occur sporadically or in the setting of hereditary polyposis syndromes, including familial adenomatous polyposis (FAP). The risk of ampullary adenomas and adenocarcinomas is increased 200- to 300-fold in such genetic polyposis syndromes (3,7). Ampullary adenomas seem to follow the adenoma-to-carcinoma sequence in progression, similar to that of colorectal cancer (8,9). The incidence of malignant transformation to carcinoma in situ or invasive carcinoma has ranged from 25% to 85% (4,10). Furthermore, endoscopic biopsy of ampullary tumours carries a 30% false-negative rate for detecting carcinoma in situ and invasive carcinoma (4,11). Thus, complete resection is mandatory at diagnosis to prevent malignant degeneration. Currently, the literature suggests that endoscopic resection (ER) in high volume centers, has similar efficacy compared to surgical ampullectomy with lower morbidity and recurrence rates in selected patients (2,3,12–33,35–44). However, the majority of these studies lack long-term follow-up data. In addition, predictors of adverse events and recurrence have not been assessed previously. In the present study, we review our experience in the management of ER for ampullary tumours in a single Canadian University Centre.

## METHODS

### Study Design

A retrospective chart review was conducted, for patients undergoing ER for ampullary tumours at The Center for Therapeutic Endoscopy and Endoscopic Oncology, St. Michael’s Hospital, University of Toronto, Toronto, Ontario, Canada over a 17-year period (between January 1999 and October 2016). Data collection included patient demographics, clinical, lesion-related and procedural data. The study was approved by the local institutional review board.

### Patients

All patients referred for ER of ampullary lesions deemed endoscopically resectable on initial assessment were included. In general, ampullary lesions (regardless of size and laterally spreading [LS] component) confined to the mucosa, with less than 1 cm intraductal growth and no evidence of invasive malignancy on endoscopic assessment (i.e., hard consistency, friable or ulcerative surface and spontaneous bleeding) were deemed suitable for ER (12,13). The preprocedural diagnostic tools including abdominal ultrasound, computed tomography scan, magnetic resonance imaging, endoscopic ultrasound and endoscopic retrograde cholangiopancreatography (ERCP) were used to determine the above mentioned criteria. The LS component was defined as any laterally spreading ampullary lesion beyond the ampullary mound.

All patients provided informed consent for the procedure after discussion of the risks and benefits. Patients receiving antiplatelet agents or anticoagulants were advised to stop these medications 3 to 7 days before the procedure as per published guidelines (45).

Clinical and endoscopic follow-up evaluation was conducted periodically as surveillance for recurrence and long-term complications.

Success was defined as complete resection of the lesion by ER with the absence of endoscopically visible and histologically proven residual lesion during a follow-up period of at least 3 months. The recorded preprocedural, procedural and postprocedural clinical variables are listed in Table 1.

**Table 1.** The data points of database for patients with ampullary lesions

Preprocedural data	Procedural data	Postprocedural data
Age	En-bloc or piecemeal endoscopic resection	Complications
Sex	Size of tumour	Postprocedural pathology
Familial adenomatous polyposis	Pancreatic stent placement	Follow-up
Clinical presentation	Biliary stent placement	Reintervention
Diagnostic tools		Referral for surgery
Preprocedural pathology		Palliative care
		Mortality

### Procedure

An experienced endoscopist performed all procedures. After an overnight fast, the procedure was performed in the endoscopy unit with fluoroscopy equipment. The majority of the patients (94.6%) received conscious sedation using midazolam and fentanyl, while the remainder received general anaesthesia, principally for patients in whom procedural intolerance was anticipated. All patients were placed in the left lateral position.

ER for ampullary lesion was performed using a therapeutic duodenoscope (Olympus TJF- 160 and TJF-180; Olympus America, Center Valley, PA). The procedure started with careful inspection of the ampulla and any LS component. The surface of the lesion was inspected for any high-risk features (ulceration, nongranular component) that may suggest invasive disease. Gentle probing of the lesion using a standard cannula usually provides an idea of the mobility and firmness of the lesion. Next, cannulation of both the bile and pancreatic ducts was attempted using standard ERCP cannula, and the ducts were partially filled with contrast to confirm the absence of intraductal extension and to define the anatomy for subsequent stent placement. We used standard, medium stiffness, braided polypectomy snares (SnareMaster; Olympus, Tokyo, Japan) 10 and 15 mm, and blended electrosurgical current (Endocut effect 3, VIO 300D; ERBE Elektromedizin, Tubingen, Germany).

For submucosal injection, we use methylene blue and saline using the Carr-Locke injection needle (US Endoscopy, Mentor, OH). In general, we injected the LS component of the lesion only to minimize the risk of perforation. However, we avoided submucosal injection of the actual ampulla since this may result in poor lifting of the center of the ampulla as it is tethered down by the biliary and pancreatic orifices, hence complicating the resection. The LS component was initially removed to isolate the ampullary lesion. The ampullary lesion was then snared from the base, and constant tension was applied to the snare loop during electrosurgery until the lesion was resected. En-bloc ER was attempted when possible, and piecemeal ER was used for LS lesions. Any immediate bleeding was stopped using snare-tip soft coagulation (effect 4, 60W - Vio 300D) or coagulation graspers (Coagrasper; Olympus).

When intraductal involvement was suspected, a small sphincterotomy was performed to expose the distal common bile duct (CBD). Next, an extraction balloon was used to try to evert the intraductal component out of the CBD for snare resection.

Prophylactic pancreatic stent (5Fr/3 cm) placement was attempted in all patients, and placed only if cannulation was successful post-ER. An abdominal x-ray was done at 10 to 14 days post-ER to assess for spontanous expulsion of the pancreatic duct (PD) stent. If the PD stent was still in place, the patient is brought back earlier for endoscopic removal.

Cholangiogram is typically done at the end of the procedure to confirm clear bile duct. Inadequate biliary drainage was defined as lack of spontaneous contrast drainage from the bile duct post-ER. Biliary stents were placed in patients with inadequate biliary drainage after resection to reduce the risk of postprocedural cholangitis. The biliary stent is typically removed during the first follow-up visit at 3 to 6 months.

The specimens were then collected using a Roth Net (US Endoscopy) and submitted for histopathology. Fluoroscopy was used to ensure correct positioning of the stents, and to rule out any extra-luminal air that may suggest perforation. In addition, over the last 4 years, Indomethacin suppository postprocedure was given routinely to all patients (unless contraindicated) for pancreatitis prophylaxis.

All patients were admitted to the hospital for observation, and kept fasting overnight while started on intravenous proton pump inhibitor therapy. Their diet was advanced the next day if no evidence of complications, and discharged once deemed safe.

**Figure 1.**
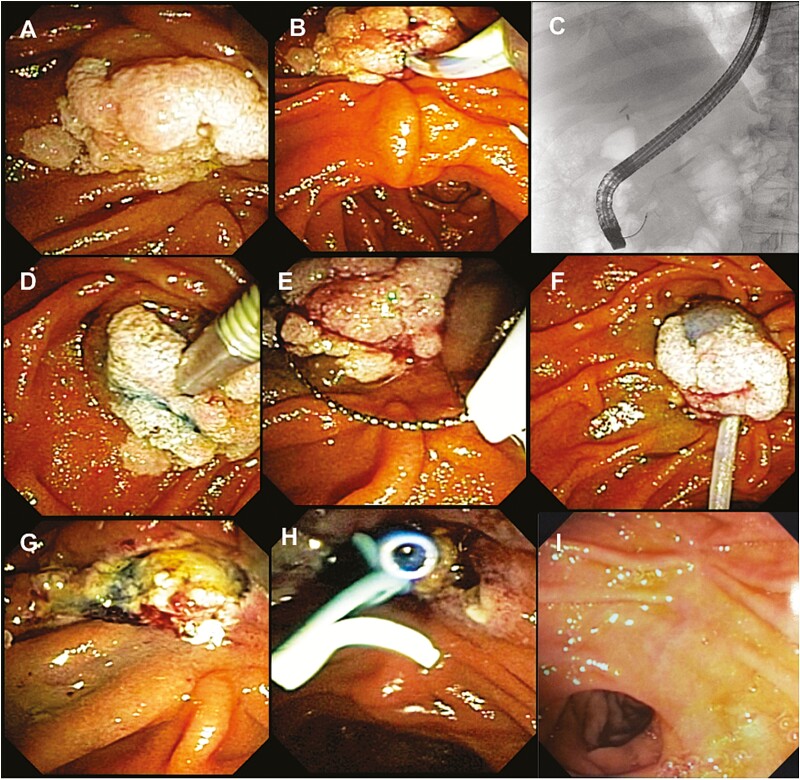
The endoscopic steps of endoscopic resection (ER) for ampullary tumours. (A) Inspection of ampullary tumour; (B, C): Cannulation of pancreatic and biliary ducts; (D) Submucosal injection; (E) Snaring; (F) The lesion is entirely entrapped by the snare; (G) En-bloc resection; (H) Stenting; and (I) Duodenal view 3 months after ER.

Follow-up was conducted periodically (initially every 3 months). Suspected adenoma recurrence was treated endoscopically with snare resection or ablative therapy (including snare-tip soft coagulation or hot avulsion using hot biopsy forceps). The adopted endoscopic steps for ampullary tumour ER are summarized in the Figure 1.

### Adverse Events Definition

Intraprocedural bleeding was defined as persistent oozing or spurting bleeding encountered during the procedure that did not stop spontaneously and required endoscopic measures to stop the bleeding. Delayed bleeding was defined as any clinically significant bleed requiring repeat endoscopy, re-hospitalization or an emergency room department visit between days 1 and 14 postprocedure. Perforation was defined as the presence of a transmural defect or radiographic evidence of free retroperitoneal or intraperitoneal air. Recurrence was defined as the presence of endoscopic and histological evidence of adenomatous tissue at the site of the resection during surveillance endoscopy.

### Outcomes

The primary outcome measure was the initial success of complete ER of the ampullary lesion. Secondary outcomes included the long-term success of ER, recurrence rates and overall morbidity and mortality of the procedure.

### Statistical Analysis

Descriptive statistics were carried out and reported as mean ± standard deviation or percentages. Inferential analyses include between group comparisons for all outcomes using the Chi-square test (or Fisher’s Exact Test) and *t*-test (or nonparametric Wilcoxon rank-sum test), where appropriate. Stepwise multivariable analyses are performed to identify risk factors for adverse events and outcomes. A statistical significance threshold of *P* < 0.05 is adopted. All analyses are performed using SAS 9.4 (SAS Institute Inc., Cary, NC).

## RESULTS

During study period, 103 patients with ampullary lesions underwent ER. All lesions were assessed with imaging and endoscopically before ER and deemed endoscopically resectable. The mean age was 62.3 years (±14.3), 52/103 (50.5%) females. The majority of the patients (85/103, 82.4%) had sporadic ampullary lesions, whereas 18 of 103 (17.6%) had FAP or attenuated FAP. The majority of the patients were symptomatic at presentation (60/103, 58.2%). The most common presenting complaint was abdominal pain (44/103, 42.7%), followed by abnormal liver enzymes (34/103, 33.0%). Mean lesion size was 20.9 mm (range 8 to 60 mm) based on pathological specimen measurement. All patients had at least 1 imaging modality performed before resection (Table 2).

**Table 2.** Patient and procedural characteristics (*N* = 103)

Characteristic (*n* = 103 patients) Patient characteristics	Value
Mean age (±SD) years	62.3 ± 14.3
Female, *n* (%)	52 (50.5)
Sporadic ampullary lesion, *n* (%)	84 (82.4)
FAP, *n* (%)	17 (16.6)
Attenuated FAP, *n* (%)	1 (1.0)
Aspirin (%)	14 (15.2)
Antiplatelet (%)	3 (3.3)
Anticoagulant (%)	10 (10.9)
Symptoms	
No symptoms, *n* (%)	43 (41.8)
Abdominal pain, *n* (%)	44 (42.7)
Jaundice, *n* (%)	13 (12.6)
Cholangitis, *n* (%)	4 (3.9)
Pancreatitis, *n* (%)	10 (9.7)
Abnormal liver enzymes, *n* (%)	34 (33.0)
Bleeding, *n* (%)	8 (7.8)
Imaging	
CT scan, *n* (%)	27 (26.2)
MRI, *n* (%)	31 (30.1)
Ultrasound, *n* (%)	17 (16.5)
EUS, *n* (%)	52 (50.5)
Procedural data	
Mass size, mm (range)	20.9 (8–60)
Resection type	
En-Bloc, *n* (%)	55 (53.4)
Piecemeal, *n* (%)	48 (46.6)
Number of pieces (±SD)	2.2 ± 2.0
Intraductal extension, *n* (%)	18 (17.5)
Sedation	
Conscious sedation, *n* (%)	97 (94.6)
General anaesthesia, *n* (%)	6 (5.4)
Sphincterotomy	
No, *n* (%)	41 (39.8)
Intraprocedural, *n* (%)	46 (44.7)
Previous sphincterotomy, *n* (%)	16 (15.5)
IPB (%)	67 (65.1)
Treatment of IPB (%)	
Thermal	57 (85.1)
Epinephrine injection	26 (38.8)
Hemostatic clips	13 (19.4)
Hemostatic powder spray	1 (1.5)
Multiple modalities to treat IPB (%)	27 (40.2)
Procedure Time (min, ±SD)	57.3 ± 24.0
Hospital stay in days, median (IQR)	3 (2–5)

CT, Computed tomography; EUS, Endoscopic ultrasound; FAB, Familial adenomatous polyposis; IPB, Intraprocedural bleeding; IQR, Interquartile range; MRI, Magnetic resonance imaging.

En-bloc resection was performed in 55 patients (53.4%). A prophylactic pancreatic stent was placed successfully in 93 of 103 (90.1%) of the patients.

Overall, a complete ER of ampullary lesions was achieved in 85 of 103 (82.5%) of the patients during the initial attempt. Among patients with benign lesions, all patients had successful ER during long-term follow-up. All patients who were found to have invasive malignancy (11 patients) were referred for surgical intervention or for palliative care. Patient, lesion and procedure characteristics are shown in Table 2.

### Pathology

#### Pre-ER Pathology

Ninety-eight patients had adenomatous lesions, including 75 (72.7%) with low-grade dysplasia (LGD), 21 (20.2%) with high-grade dysplasia (HGD) and 3 (3.0%) with intramucosal carcinoma.

#### Post-ER Pathology

Ninety-one patients had confirmed adenomatous lesions with LGD confirmed in 46 patients (44.0%), whereas HGD was found in 31 patients (30.0%) and intramucosal carcinoma in 7 patients (7.0%). Furthermore, invasive malignancy was identified in 11 patients (11.0%). The preprocedural and postprocedural pathology results are summarized in Table 3.

**Table 3.** Pathological characteristics of resected lesions

Pre-ER pathology	*N* (%)
Adenoma (villous)	16 (15.5)
Adenoma (tubular)	62 (59.8)
Adenoma (tubulovillous)	20 (19.6)
Neuroendocrine tumour	1 (1.0)
Normal intestinal mucosa	2 (2.1)
Inflammatory	2 (2.1)
Pre-ER dysplasia/cancer	*N* (%)
LGD	75 (72.7)
HGD	21 (20.2)
IMC	3 (3.0)
No dysplasia	4 (4.0)
Post-ER pathology	*N* (%)
Adenoma (villous)	7 (7.1)
Adenoma (tubular)	66 (64.0)
Adenoma (tubulovillous)	18 (17.7)
Ganglioneuroma	1 (1.0)
Neuroendocrine tumour	3 (2.4)
Normal Intestinal Mucosa	7 (6.7)
Inflammatory	1 (1.0)
Post-ER dysplasia/cancer	*N* (%)
LGD	46 (44.0)
HGD	31 (30.0)
Malignant	11 (11.0)
No dysplasia	8 (8.0)
IMC	7 (7.0)

ER, Endoscopic resection; HGD, High-grade dysplasia; IMC, Intramucosal carcinoma; LGD, Low-grade dysplasia.

### Adverse Events

#### Delayed Bleeding

The most common adverse event was delayed bleeding (22 patients, 21.4%; Table 4). Among these patients, 10 patients (45.5%) required endoscopic intervention to stop the bleeding. Only eight patients (36.4%) required blood transfusions. None required radiological or surgical interventions to stop the bleeding.

**Table 4.** Postprocedure complications

Complication	*N* (%)
Delayed Bleeding	22 (21.4)
Endoscopic treatment	10 (45.5)
Blood transfusion	8 (36.4)
Interventional radiology	0 (0)
Surgery	0 (0)
Acute pancreatitis	16 (15.5)
Mild	15 (93.8)
Severe	1 (6.2)
Perforation	6 (5.8)
Conservative	5 (83.3)
Surgery	1 (16.7)
Cholangitis	4 (3.9)
Ampullary stenosis	12 (15.6)
Endoscopic dilation success	12 (100)
Surgery	0 (0)
Procedure-related mortality	0 (0)

#### Pancreatitis

Acute pancreatitis occurred in 16 patients (15.5%). The majority of the patients had mild acute pancreatitis (93.8%). There was no significant difference in the rate of pancreatitis between patients who had a PD stent (93 patients) and those who did not (10 patients), (16.1% versus 10%, *P* = 1.00).

#### Perforation

Retroperitoneal perforation occurred in six patients (5.8%) with only one patient requiring surgery to manage the perforation.

#### Cholangitis

Overall, four patients (3.9%) had postprocedure cholangitis; all were treated conservatively.

#### Ampullary Stenosis

During follow-up, 12 patients (15.6%) developed ampullary stenosis which was treated successfully by endoscopic dilation.

**Figure 2.**
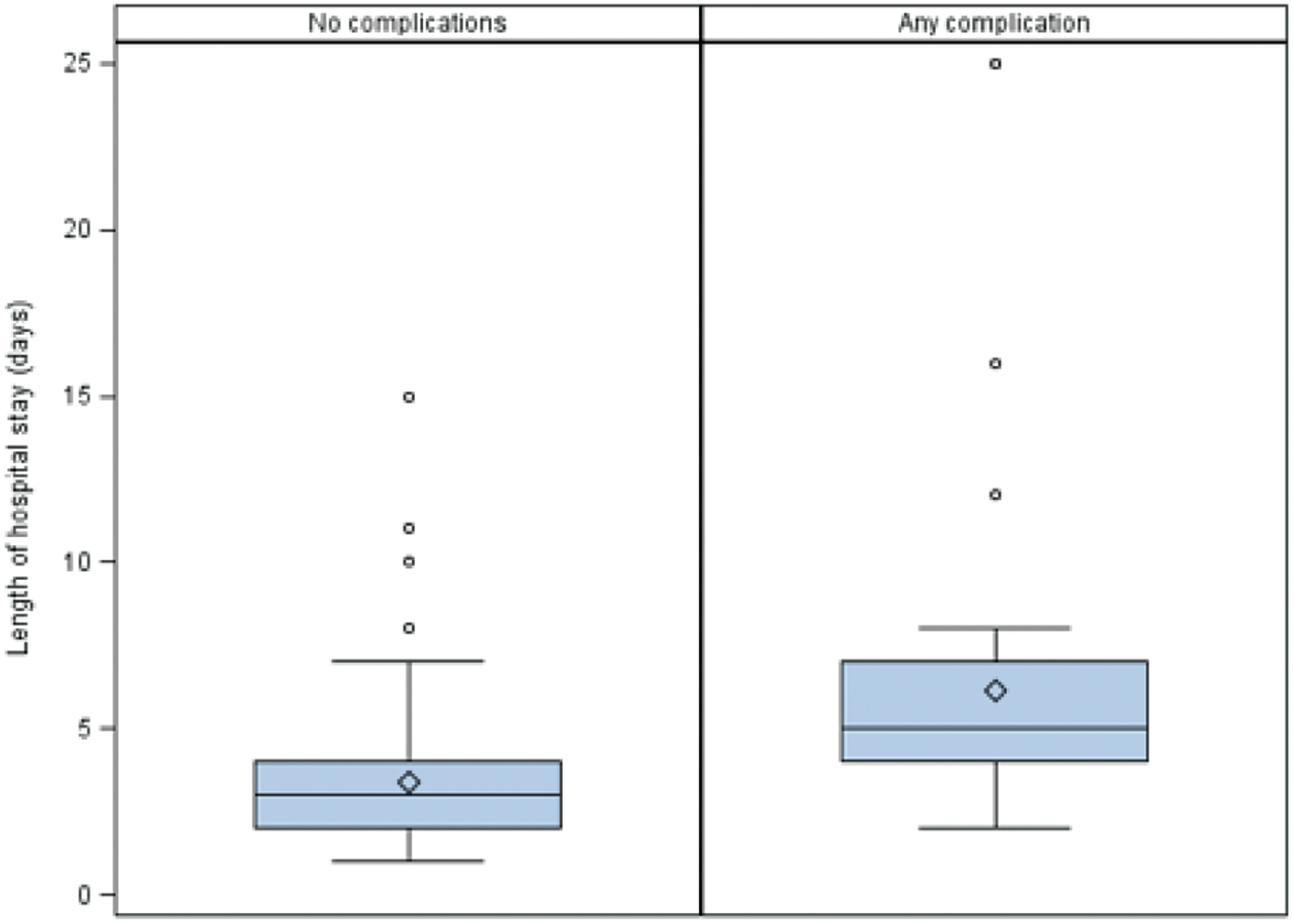
Duration of hospital stay among patients with and without complications.

Among patients who suffered a complication, the median hospital stay was significantly longer compared to patients with no complications (3 versus 5 days, *P* < 0.0001; Figure 2). There was no procedure-related mortality.

### Follow-up

The majority of the patients had at least one follow-up visit (77/103, 75%). The median number of days from the procedure to the first follow-up visit was 127 days (interquartile range 93 to 182). The longest follow-up was 15 years postprocedure.

Among the patients with at least one follow-up visit (77 patients), the majority (47 patients, 61%) had no recurrence and were considered cured from their index procedure. The other 30 patients (39%) had some ampullary lesion recurrence during follow-up. The majority of recurrences were seen during the first follow-up visit (73.3%). All recurrences were treated successfully endoscopically. Of note, some recurrences were only detected during long-term follow-up (Table 5).

**Table 5.** Timing of initial recurrences detected during surveillance

	Days after index procedure Median (IQR)	Recurrence (*n*)	Recurrence (%)
First surveillance	127 (93–182)	22	73.3%
Second surveillance	354 (222–523)	3	10%
Third Surveillance	591 (390–1148)	3	10%
Fourth Surveillance	932 (449–1181)	1	0.3%
Fifth Surveillance	1095 (309–2169)	1	0.3%
Sixth Surveillance	1715 (1345–3383)	0	0%

### En-bloc Versus Piecemeal Resection

Recurrences occurred significantly more frequently if piecemeal resection was used compared to en-bloc resection (54.3% versus 26.2%, respectively, *P* = 0.0118). There were no differences in complications rate (Table 6).

**Table 6.** En-bloc versus piecemeal resection adverse events

Resection type	En-bloc	Piecemeal	*P*-value
Number	55 (53%)	48 (47%)	
Mean size (mm)	17.3	24.9	0.0004
Intraprocedural bleeding	35 (63.6%)	32 (66.6%)	0.751
Delayed bleeding	8 (14.5%)	14 (29.2%)	0.072
Pancreatitis	9 (16.4%)	7 (14.6%)	0.805
Perforation	3 (5.5%)	3 (6.3%)	0.865

### Predictors of Adverse Events

Using univariable and multivariable analyses, the use of antiplatelet or anticoagulant agents, tumour size, number of resection pieces and procedure time were not associated with increased risks of adverse events (Table 7).

**Table 7.** Univariable and multivariable analysis of predictors of adverse events

Variable	No complications	Complications	*P*-values
Aspirin, antiplatelet or anticoagulant use (%)	26.3	25.8	0.9652
Mass size (mm)	21.3 ± 11.4	20.0 ± 10.8	0.4142
Number of ampullectomy pieces	2.1 ± 2.1	2.4 ± 1.8	0.1259
Procedure time (minutes)	57.7 ± 24.3	56.5 ± 23.7	0.6755
Odds ratio estimates			
Effect	Point estimate	95% Wald confidence limits	
Aspirin, antiplatelet or anticoagulant	1.504	0.513	4.415
Mass size (mm)	0.970	0.917	1.025
Number of resection pieces	1.163	0.905	1.494
Procedure time (minutes)	0.999	0.975	1.023

## Discussion

Historically, ampullary lesions have been treated surgically using Whipple’s procedure or transduodenal ampullectomy (2–4,10,12,13). Pancreatoduodenectomy is associated with a higher morbidity (50% to 60%) and mortality (0% to 9%) compared with transduodenal ampullectomy (morbidity, 14% to 27%; mortality 0% to 4%) (4,46). However, recurrence rates are high (30%) with transduodenal excision, requiring close endoscopic surveillance after surgery (47).

Currently, ER of ampullary lesions represents a viable alternative to surgical treatment in selected patients. ER was first reported by Suzuki et al. in 1983 (48) and the first large case series was reported by Binmoeller et al. in 1993 (15). Since then, many other series have reported success rates for ER ranging from 29% to 100%, with an overall success rate of about 79%. The recurrence rates of ampullary adenomas after ER range from 0% to 33%, with an overall incidence of about 12%. The overall morbidity rate is about 20% (5% to 56%) with a mortality ranging from 0% to 7% (16–33,35–44).

The present study reports similar outcomes for ER of ampullary lesions to that of other published studies. The success rates for ER in the long-term were close to 90% in this cohort with the remainder of patients being referred for surgery because of a diagnosed invasive malignancy. The recurrence rates of ampullary tumours after successful ER was 39%, which is higher compared to previously published reports (10). This is likely related to longer follow-up in our cohort with many patients experiencing delayed recurrence (26.7% of recurrences identified at/after 1 year postindex ER). All these patients were treated successfully endoscopically during follow-up. Therefore, long-term follow-up of patients post-ER is crucial to detect and manage late recurrences. An important finding of this study is the recurrence rate was significantly higher among patients who had piecemeal resection compared to en-bloc resection (54.3% versus 26.2%, *P* = 0.0118) with similar safety profile. This highlights the importance of achieving en-bloc resection whenever possible to reduce the risk of recurrence.

Another important finding was the postprocedural pathology percentage of invasive cancers (11%) not detected despite careful preresection assessment. These data support the previous observation of a significant false-negative rates of forceps biopsy specimens for detecting both carcinoma in situ and invasive carcinoma (4,11,49). Therefore, complete resection of the tumour is mandatory at diagnosis to confirm diagnosis and prevent malignant degeneration.

Even though complications were relatively common, most complications were mild and managed nonsurgically. Our study demonstrates that having any complication post-ER significantly prolongs hospital stay but is unlikely to bear long-term consequences. The most important complications were delayed bleeding (21.4%), pancreatitis (15.5%), perforation (5.8%) and cholangitis (3.9%), which were similar to previously reported morbidity rates in other series (16–33,35–44). Acute pancreatitis is one of the most common complications after ER. In our study, all but one of the patients developing pancreatitis had received a prophylactic pancreatic duct stent. It is difficult retrospectively to address the exact factors contributing to postampullectomy pancreatitis, including the choice of electrocautery setting or other intraprocedural manipulations. Furthermore, our study was not powered to address benefits of prophylactic pancreatic stent insertion at reducing the risk of postampullectomy pancreatitis. However, a randomized study has previously shown clear benefits attributable to prophylactic pancreatic stent insertion (28), hence it should be standard of care. In our study, there was no mortality related to the procedure. Death is rare and has been reported in two patients previously (17,26).

In the long-term, papillary stenosis was the most frequent complication (15.6%); all were treated successfully by endoscopy. These findings are similar to previous reports (range 0% to 10%) (16–33,35–44).

The main strengths of our study are the large number of patients included and the long-term follow-up. To our knowledge, this study is the first to confirm that en-bloc resection of ampullary tumours significantly reduces recurrence rates. In addition, we were able to show that a significant number of recurrences are only encountered during long-term follow-up necessitating continued surveillance of such patients.

There were several potential limitations to our study. First, this is a retrospective study, with all its drawbacks including missing data; indeed in 26 patients, the follow-up was not available because they were followed in others centers. Also, due to unavailable information, we were not able to adjust the results according to the presence of a LS component of the ampullary tumour, which may potentially explain the higher recurrence rate with piecemeal resection.

## Conclusions

In summary, the present study results confirm the safety and efficacy of ER of ampullary lesions when performed by experienced endoscopists with acceptable morbidity and no mortality. Even though complications are relatively common, they can easily be managed endoscopically. En-bloc resection may significantly reduce recurrence rates. Long-term follow-up is needed as delayed recurrences are common.

## Notes

**Author’s Contribution**

A.A.: Study concept and design, acquisition of data, analysis and interpretation of data, drafting and revision of the manuscript; A.E.: Study concept and design, acquisition of data, analysis and interpretation of data, drafting and revision of the manuscript; M.M.: Acquisition of data, drafting and revision of the manuscript; M.M.: Analysis and interpretation of data, drafting and revision of the manuscript; I.S.: Acquisition of data, drafting and revision of the manuscript; C.S.: Acquisition of data, drafting and revision of the manuscript; M.C.: Acquisition of data, drafting and revision of the manuscript; J.M.: Study concept and design, analysis and interpretation of data, revision of the manuscript; P.K.: Study concept and design, analysis and interpretation of data, revision of the manuscript; A.B.: Study concept and design, analysis and interpretation of data, revision of the manuscript; G.R.M.: Study concept and design, analysis and interpretation of data, drafting and revision of the manuscript. All authors approved the final version of the manuscript.

A.A., A.E., M.M., M.M., I.S., M.C., C.S., J.M., P.K., A.B. and G.R.M. have no financial relationships relevant to this publication to disclose.

This manuscript has not been published previously in print or electronic format and is not under consideration by another publication or electronic medium.
